# Chromosomal plasticity and evolutionary potential in the malaria vector *Anopheles gambiae *sensu stricto: insights from three decades of rare paracentric inversions

**DOI:** 10.1186/1471-2148-8-309

**Published:** 2008-11-10

**Authors:** Marco Pombi, Beniamino Caputo, Frederic Simard, Maria A Di Deco, Mario Coluzzi, Alessandra della Torre, Carlo Costantini, Nora J Besansky, Vincenzo Petrarca

**Affiliations:** 1 Sezione di Parassitologia, Dipartimento di Scienze di Sanità Pubblica, Università di Roma "Sapienza", P.le Aldo Moro 5, 00185 Roma, Italy; 2 Istituto Pasteur – Fondazione Cenci Bolognetti, P.le Aldo Moro 5, 00185 Roma, Italy; 3 Institut de Recherche pour le Développement, Unité de Recherche 016, and Institut de Recherche en Sciences de la Santé, BP 545 Bobo Dioulasso, Burkina Faso; 4 Institut de Recherche pour le Développement, Unité de Recherche 016, and Organisation de Coordination pour la lutte contre les Endémies en Afrique Centrale, BP 288 Yaoundé, Cameroon; 5 Eck Family Center for Global Health and Infectious Diseases, Dept. of Biological Sciences, University of Notre Dame, Notre Dame, IN 46556, USA; 6 Dipartimento di Genetica e Biologia Molecolare, Università di Roma "Sapienza", P.le Aldo Moro 5, 00185 Roma, Italy

## Abstract

**Background:**

In the *Anopheles gambiae *complex, paracentric chromosomal inversions are non-randomly distributed along the complement: 18/31 (58%) of common polymorphic inversions are on chromosome arm 2R, which represents only ~30% of the complement. Moreover, in *An. gambiae *sensu stricto, 6/7 common polymorphic inversions occur on 2R. Most of these inversions are considered markers of ecological adaptation that increase the fitness of the carriers of alternative karyotypes in contrasting habitats. However, little is known about the evolutionary forces responsible for their origin and subsequent establishment in field populations.

**Results:**

Here, we present data on 82 previously undescribed rare chromosomal inversions (RCIs) recorded during extensive field sampling in 16 African countries over a 30 year period, which may shed light on the dynamics of chromosomal plasticity in *An. gambiae*. We analyzed breakpoint distribution, length, and geographic distribution of RCIs, and compared these measures to those of the common inversions. We found that RCIs, like common inversions, are disproportionately clustered on 2R, which may indicate that this arm is especially prone to breakages. However, contrasting patterns were observed between the geographic distribution of common inversions and RCIs. RCIs were equally frequent across biomes and on both sides of the Great Rift Valley (GRV), whereas common inversions predominated in arid ecological settings and west of the GRV. Moreover, the distribution of RCI lengths followed a random pattern while common inversions were significantly less frequent at shorter lengths.

**Conclusion:**

Because 17/82 (21%) RCIs were found repeatedly at very low frequencies – at the same sampling location in different years and/or in different sampling locations – we suggest that RCIs are subject mainly to drift under unperturbed ecological conditions. Nevertheless, RCIs may represent an important reservoir of genetic variation for *An. gambiae *in response to environmental changes, further testifying to the considerable evolutionary potential hidden within this pan-African malaria vector.

## Background

Chromosomal paracentric inversions are mutations where part of a chromosome, not including the centromere, has been reversed with respect to a standard orientation of reference. These inversions have now been described from a diversity of species, including humans [1], but historically were most readily observed in dipteran groups such as midges, blackflies, fruitflies and anopheline mosquitoes, where the presence of giant (polytene) chromosomes facilitates detection and analysis of inversions [2,3].

Theoretical and empirical studies in natural populations suggest that chromosomal inversion polymorphisms can be maintained by selection acting to preserve beneficial allelic content from recombination between alternative arrangements, aided by reduced gene exchange in heterokaryotypes [2,4-10]. This mechanism is hypothesized to have played a central role in the ecotypic differentiation and speciation events represented by the *Anopheles gambiae *complex [4,5,11,12], an African group of closely related mosquitoes that contains two of the most significant vectors of human malaria. Although virtually morphologically identical at all developmental stages, most species in the complex are distinguished by at least one fixed inversion difference. In the nominal species and most important vector *Anopheles gambiae *sensu stricto (hereafter simply *An. gambiae*), polymorphic inversions on chromosome 2 are distributed nonrandomly with respect to environmental variables such as aridity, leading to temporally stable geographic clines of inversion frequencies in different parts of Africa, regular cycling of inversion frequencies with respect to rainy and dry seasons, and local ecological and behavioural heterogeneities. Collectively, these inversions are considered markers of local ecological adaptation that increase the suitability of alternative karyotypes in contrasting habitats. This view is based on observations showing higher frequencies of inverted arrangements in arid Savannas rather than in forest areas of equatorial Africa, and in indoor rather than in outdoor collected samples, where the saturation deficit is generally lower [5,11,13-15].

Despite the longstanding nature of these observations and their bearing on the genetic flexibility and evolutionary potential of *An. gambiae*, little is yet known about the forces responsible for the origin and establishment of chromosomal inversions in *An. gambiae *populations. Some insight into the origin of inversions can be gained through molecular cloning of the breakpoints of already established inversions, and available evidence suggests a role for repetitive DNA in the form of transposable elements or segmental duplications [16-18]. Here we adopt a different approach focused on previously undescribed rare chromosomal inversions (RCIs) of *An. gambiae*, recorded during extensive karyotyping of field populations sampled in many African countries over a thirty year period. Given three stages in the evolutionary history of an inversion (origin, establishment and maintenance), we consider RCIs to be early in the process of establishment: present in several copies in the population and potentially subject to selection and drift [9]. We measured the breakpoint locations, length and geographic distribution of 82 RCIs, and compared these measures to those of the common inversion polymorphisms. We find that RCIs, like common inversions, are disproportionately clustered on chromosome arm 2R. However, unlike the common *An. gambiae *inversions, their frequency does not differ between ecological zones or between East and West Africa. We suggest that RCIs represent a reservoir of genetic variation in this pan-African malaria vector.

## Methods

### Sampling, species identification and chromosomal scoring

Samples of *An. gambiae *were collected at different times of the year in 16 Afrotropical countries from 1975 to 2006 (Additional file 1: Table S1). The majority of sampling consisted of daytime indoor-resting catches by manual or insecticide spray collections. Polytene chromosomes from ovarian nurse cells of half-gravid female mosquitoes were prepared as described previously [19,20]. Species of the *An. gambiae *complex were identified by microscopic examination of sets of species-specific fixed inversions [5]. The M and S molecular forms of *An. gambiae *were not identified in most samples, as the majority of the data were obtained before their recognition [21]. Accordingly, data analyses were not stratified by molecular form.

Paracentric inversions that differed from previously characterized inversions commonly observed in this species [11], hereafter called RCIs, were recorded in a database. The localization of each inversion breakpoint was determined with reference to the *An. gambiae *polytene chromosome map (published as a poster in Science 298, 4 October 2002 by M. Coluzzi and V. Petrarca; http://www.sciencemag.org/feature/data/mosquito/index.dtl#poster) by further dividing each subdivision into 10 parts (from 0 to 9) [11] (Additional file 2: Figure S1), operatively defined here as *infra-divisions*. For example, the breakpoint of an inversion that is recorded in the 3^rd ^part of the sub-division 17B is defined as standing in 17B2.

Karyotyping of samples collected from Cameroon in 2005 and the Senegambia area in 2005–2006 was conducted with the specific intention of recording the frequency of RCIs, noting sample sizes from all localities in which RCIs were present as well as absent. Although RCIs were recorded whenever they were detected in all other collections, this was not the goal of those collections, thus corresponding information regarding whole karyotype, sample sizes, geographic coordinates, and year of collection was not always available with respect to individual sampling localities (V. Petrarca, unpublished data), and are therefore missing from Table S1. Importantly, although site-specific information was incomplete, information about overall sample sizes of collections performed over time and/or larger geographic areas was preserved.

### Expected distribution of inversion tract lengths and breakpoints

Assuming that chromosomal breaks are random and independent, the expected distribution of inversion tract lengths was generated using the Nadeau-Taylor random breakage model [22]. Under this model, expected inversion tract lengths approximate an exponential distribution with density function *f(x) *= (1/*L*)*e*^(-*x*/*L*)^, where L is the average length of all tracts [23]. The observed inversion length was expressed as a percentage of the length of 2R, estimated by dividing the interval length between breakpoints by the whole length of 2R (see below).

As the molecular structure of the RCI breakpoints is undetermined, it was not possible to localize the breakpoints at the DNA sequence level relative to the *An. gambiae *reference genome. Instead, analysis of the spatial distribution of inversion breakpoints along the polytene complement was conducted at the cytological level. We avoided reference to the polytene divisions of the cytogenetic map, as these divisions are unequal in length. To achieve a more uniform partitioning of the chromosome complement, we employed intervals of microscopically similar length, although we are aware that fragments of polytene chromosomes of equal length do not match strictly equal lengths of DNA base pairs, due to local variations in chromatin quantity [24]. As polytene chromosomes could not always be partitioned in an integer number of intervals of fixed length, the lengths of each interval were adjusted to allow each chromosome arm to be subdivided into an integer number of intervals. In consequence, we defined 66 intervals for the entire chromosomal complement: 20 for chromosomal arm 2R, 15 for 2L, 14 for 3R, 12 for 3L, and 5 for the X-chromosome.

## Results

**Figure 1:**
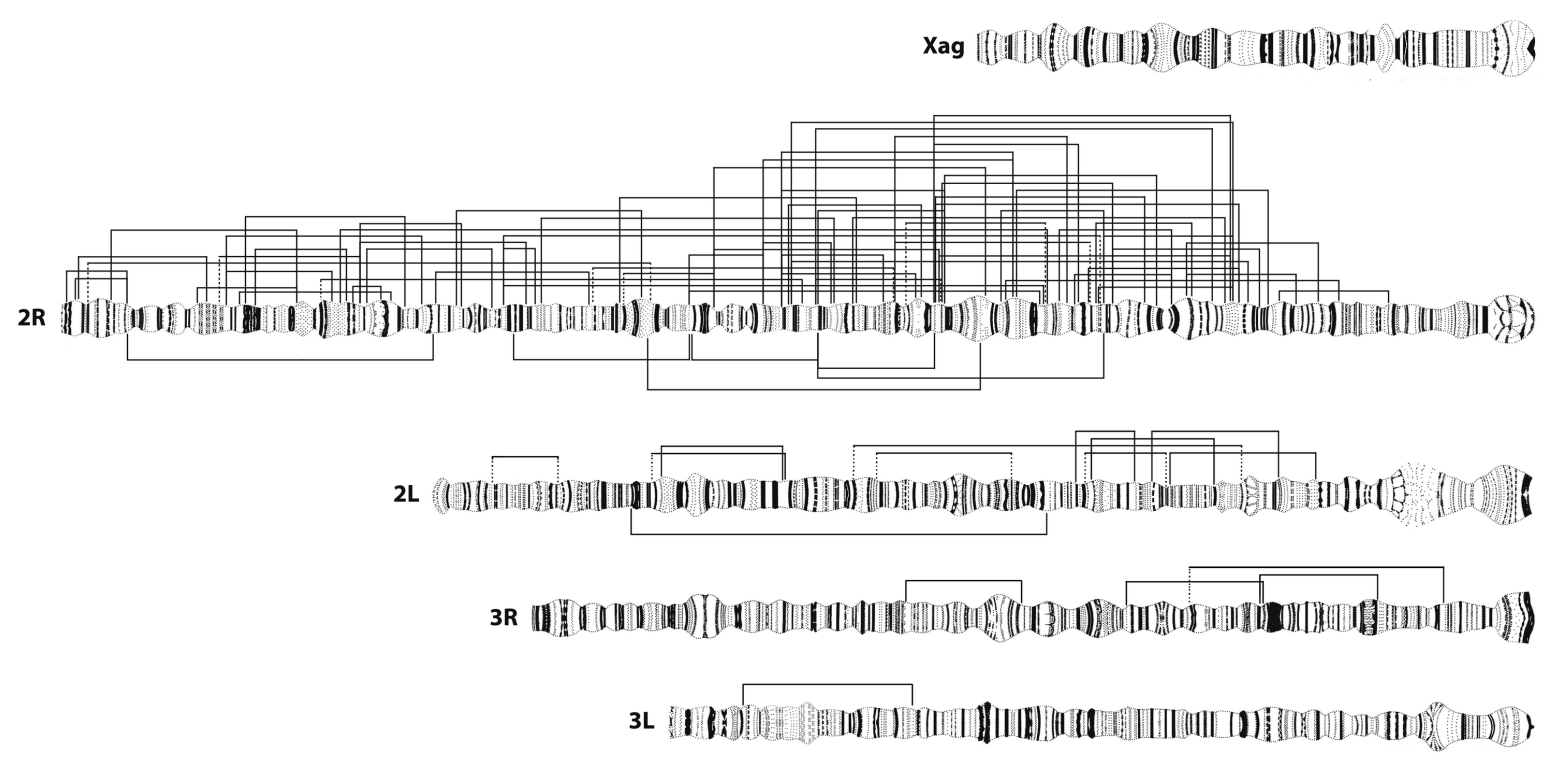
**Paracentric chromosomal inversions of *Anopheles gambiae *sensu stricto**. Location of 82 rare chromosomal inversions (above) and 7 common chromosomal inversions (below) on the *An. gambiae *polytene chromosome complement. Dotted lines indicate breakpoints that could not be unambiguously located to a single infradivision.

Among > 7,300 *An. gambiae *half-gravid females analyzed from 16 Afrotropical countries over a thirty year period, 82 previously undescribed RCIs were recorded in 160 specimens (Fig. 1; Table S1; Additional file 3: Table S2). Of these 82, 17 (21%) were found repeatedly, at the same sampling location across different years and/or in different sampling locations – in some cases different countries. All except two of the remaining 65 inversions were found only once in single specimens; the two exceptions were found in two or four specimens from the same sample.

The frequency of observed RCIs could be estimated most reliably for samples collected in Cameroon (2005) (Simard F, Ayala D, Kamdem GC, Pombi M, Etouna J, Ose K, Fosting JM, Fontenille D, Besansky N and Costantini C, unpublished) and in the Senegambia region (2005–2006) (B. Caputo, D. Nwakanma, M. Jawara, I. Dia, L. Konate, M. Coluzzi, V. Petrarca, D.J. Conway, A. della Torre, unpublished). For other collections, documentation of sample sizes from individual localities (including those in which no RCI was detected) is incomplete. The frequency of occurrence was 13/2,080 RCIs/individuals (0.6%; 95% confidence interval = 0.4%–1.1%) in samples collected from 225 sites in Cameroon and 6/1,608 (0.4%; 95% CI = 0.2%–0.8%) in samples collected in 35 sites from the Senegambia region.

The overall data provide information about the frequency distribution of RCI tract lengths, the pattern of distribution of their breakpoints on the cytogenetic map, and their geographic distribution in *An. gambiae*. Below, we compare these patterns to those observed for the common chromosomal inversions in this and other species.

### Length distribution of RCIs

**Figure 2:**
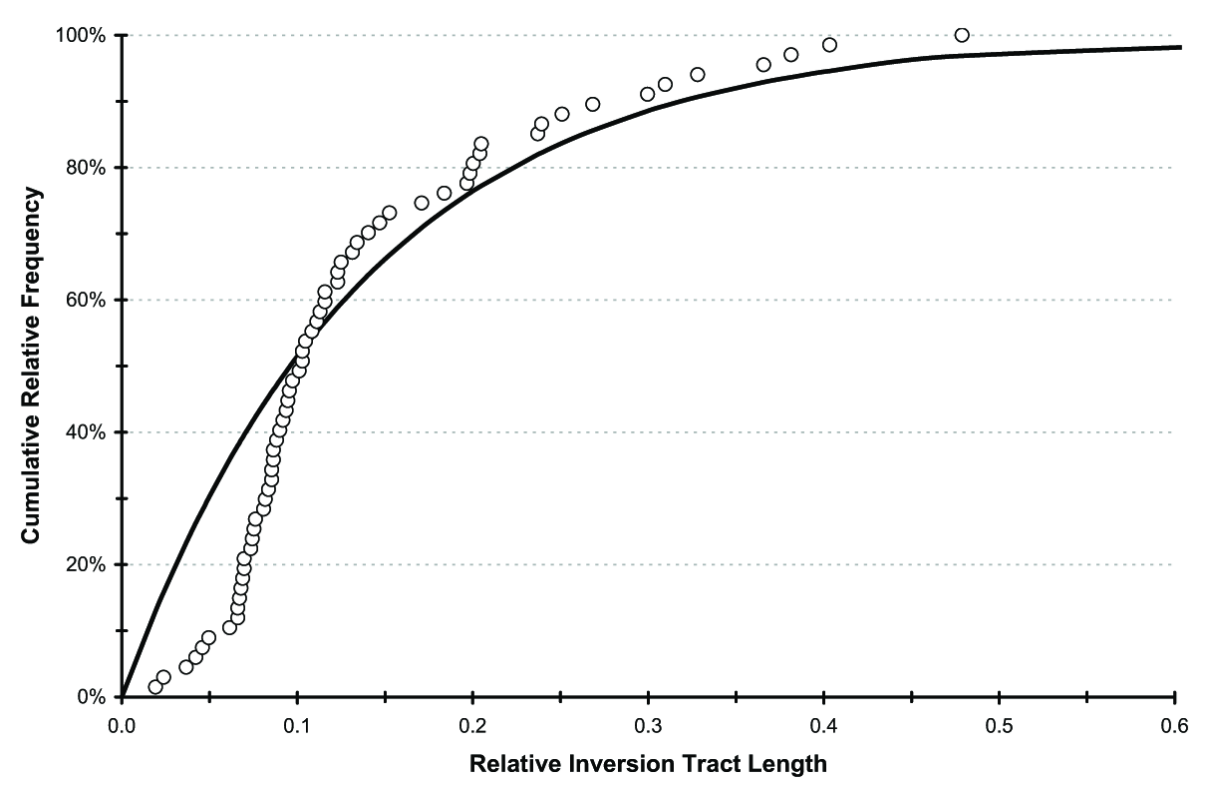
**Length distribution of rare chromosomal inversions for chromosome arm 2R of *Anopheles gambiae *sensu stricto**. Distribution expected from the random breakage model (solid line), plotted along with the observed distribution of relative tract lengths (circles).

If chromosomal segments break at random, the distribution of observed inversion tract lengths should follow a random pattern. We tested this hypothesis on chromosome arm 2R, where most (67/82) RCIs and most (6/7) common inversions are observed. Given 67 inversions, we simulated their length distribution if they were generated under a random breakage model [22], and compared this to the observed length distribution of RCIs (Fig. 2). The observed distribution departed significantly from that expected under a model of random breakage (Kolmogorov-Smirnov one-sample test, P < 0.01) due to a deficit of shorter lengths. Although this result broadly agrees with emerging evidence against the random breakage model [25-30], it should be treated with caution as the sample of RCIs is biased to an unknown extent toward larger inversion tracts: small inversions are difficult or impossible to observe microscopically, particularly when fixed. In fact, Ranz *et al. *[31] mapping by in situ hybridization 33 DNA clones containing protein-coding genes in *Drosophila repleta *and *D. buzzatii*, showed extensive reorganization via paracentric inversions, including short ones that had gone undetected with the classical polytene chromosome analysis.

To assess whether the length distribution of common inversions differs from that of RCIs, we compared the distributions observed for the 67 RCIs and the six common inversions on 2R, and found a highly significant difference (Kolmogorov-Smirnov two-sample test, P < 0.001). Extending the comparison to the 77 RCIs and seven common inversions on all of chromosome 2 did not alter the result (Kolmogorov-Smirnov two-sample test, P < 0.001). Both comparisons revealed that common inversions were less frequent at shorter lengths, relative to the length distribution of RCIs. Although these results should be interpreted with caution given the small sample size of common inversions, they agree with previous work on *Drosophila *species: rare inversions are predominantly small compared to the evolutionarily successful ones, which were predominantly intermediate in length [9,32]. Therefore, assuming that common polymorphic inversions are subject to balancing selection (in distinction to RCIs), the paucity of shorter common inversions may reflect their smaller selective advantage due to the capture of fewer genes.

### Breakpoint distribution of RCIs

**Figure 3:**
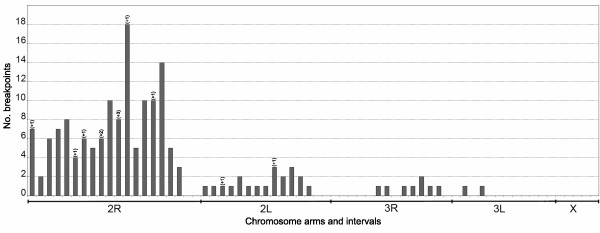
**Frequency distribution of rare chromosomal inversion breakpoints of *Anopheles gambiae *sensu stricto**. Frequency distribution of RCIs breakpoints in intervals along the chromosome complement (20 for chromosome arm 2R, 15 for 2L, 14 for 3R, 12 for 3L, and 5 for X-chromosome) of *An. gambiae*. The corresponding chromosome arms (indicated beneath the plot as a horizontal line) are presented with telomeres at left and centromeres at right. Numbers above the bars indicate the number of breakpoints that must be added to account for the common inversions.

Eighty-two percent of RCI breakpoints were found on chromosomal arm 2R (134), 12% on 2L (20), 5% on 3L (8), and 1% on 3R (2). No RCIs were found on the X chromosome. A non-uniform distribution of RCI breakpoints across the polytene complement is apparent from visual inspection of Fig. 3, in which breakpoints observed per chromosome interval are plotted. This was confirmed by a goodness of fit test comparing the observed numbers of breakpoints on each of five chromosome arms to the number expected if all 164 breakpoints were distributed uniformly across the five arms (χ^2 ^= 210.3, df = 4, P < < 0.001). Deviation from expectation was due to a large excess of RCI breakpoints on 2R and a deficit of breakpoints on the other arms, particularly those of chromosome 3 and the X.

As a second step, we considered the breakpoint distribution on chromosome 2R and tested for deviation from a uniform distribution of the 134 breakpoints observed across the intervals on this arm. There was a significant deviation (χ^2 ^= 53.8, df = 19, P < < 0.001), owing mostly to a large excess of breakpoints in intervals 12 and 16 (corresponding to cytogenetic subdivisions 14C4-15A2 and 16D4-17C0) and the absence of breakpoints in the two intervals nearest the centromere, as seen in Fig 3.

The extent to which breakpoints are non-randomly clustered on chromosome arm 2R is not captured completely at the interval level of resolution. This is emphasized by the sharing of cytologically identical breakpoints between at least two different (and presumably independent) inversions, of which there were 21 separate instances, particularly involving subdivisions 14D-15A and 16D-17B (Table S2). Thus, to test for non-independence of breakpoint distribution at the highest possible level of resolution on chromosome arm 2R, we used the infra-divisional map location of RCI breakpoints given in Table S2 (roughly the resolution of band/interband). The 540 infradivisions of chromosome arm 2R were classified as containing one, two, three, four or no breakpoints; these were compared to the number expected according to a Poisson distribution (Table 1). The goodness of fit test confirmed that breakpoints coincided at a much higher rate than expected by chance (χ^2 ^= 13.1; df = 2; P = 0.0014).

**Table 1:** Chi-square analysis of coincident rare chromosomal inversion breakpoints on chromosome arm 2R of *Anopheles gambiae *sensu stricto

	No. Infradivisions			
	
Breakpoints per Infradivison	Observed		Expected	
0	445		430.80	
1	74		97.33	
2	17	21	10.99	11.87
3	2	21	0.83	11.87
4	2	21	0.05	11.87

Χ^2^_2 _= 13.08; *P *= 0.0014

The nonrandom pattern of distribution of RCI breakpoints mirrors that of the common polymorphic inversions in the *An. gambiae *complex in three respects. First, in *An. gambiae*, common inversions are absent from chromosomes 3 and X, and RCIs are significantly underrepresented on these chromosomes. Second, in both the *An. gambiae *complex and its nominal species there is a striking overrepresentation of common polymorphic inversions on chromosome arm 2R (*e.g. *18 of 31 (58%) in *An. gambiae *sensu lato, and 6 of 7 (86%) in *An. gambiae*) [5,11], just as 67 of 82 (82%) RCIs are located on this arm, which represents less than 30% of the polytene complement. It also bears mention that the central part of 2R is subsumed not only by polymorphic inversions, but also by some fixed inversions that differentiate members of the species complex. Third, RCIs and common inversions may have cytologically coincident breakpoints on 2R (14/67: 21%).

### Ecological and geographic distribution of RCIs

In *An. gambiae *populations of West and Central Africa, there are clines in both the number of common inversion polymorphisms and in the relative frequency of their alternative arrangements that are correlated with environmental and ecological factors. In humid forested regions, polymorphisms are nearly absent and the standard (uninverted) arrangements prevail, whereas Guinea and Sudan savanna populations are characterized by many highly polymorphic inversions [5,11,14]. A similar pattern would be anticipated for RCIs if their origin and/or establishment is more likely in savanna versus forested regions. To examine the frequency of RCIs in different ecological settings, we stratified the 225 villages sampled during 2005 in Cameroon according to the country's three main biomes: forest, Guinea savanna and Sudan savanna. Whereas significant heterogeneity between biomes was observed in the case of common inversions (χ^2 ^= 155.3; df = 2; P < 0.001), no significant differences in the occurrence of RCIs were found among the three biomes (χ^2 ^= 3.8, df = 2, P = 0.15), suggesting that – at least in Cameroon – RCIs have the same probability of origin and/or establishment in different ecological contexts.

At a continental scale, the distribution of the common inversion polymorphisms in *An. gambiae *on opposite sides of the Great Rift Valley also differs. East of the Rift Valley, populations contain fewer inversion systems and lower levels of polymorphism than western populations [11,33-38]. To assess whether levels of RCI polymorphism reflect this same trend, we compared the frequency of RCIs on both sides of the Rift Valley. Toward this end, we used total sample sizes from multiple collections east and west of the Rift Valley. In populations on the western side, 77 RCIs were found in a total of ~45,000 specimens (0.17%); on the eastern side, 5 RCIs were observed among ~2,000 specimens (0.25%) [11,33-38], and Petrarca, unpublished data). The difference between RCI frequencies on either side of the Rift Valley is not significant (χ^2 ^= 0.47, df = 1, P = 0.49). Comparable results were obtained for a second member of the *An. gambiae *complex, the sibling species *An. *arabiensis. In this species, just as for *An. gambiae*, common inversion polymorphisms predominate west of the Rift Valley [11,35], but RCI frequencies are not significantly different on either side of the Rift Valley: 21 RCIs among ~14,000 specimens (0.15%) in the west versus 3 RCIs among ~1,850 specimens (0.16%) to the east (χ^2 ^= 0.03, df = 1, P = 0.86; [35]; Petrarca, unpublished data).

## Discussion

Longstanding evidence from cytological studies together with more recent evidence from comparative whole genome sequence analysis is revealing common themes concerning the distribution patterns of chromosomal rearrangements in eukaryotic genomes [3,39]. Within closely related groups of *Drosophila *and *Anopheles*, certain species carry abundant inversion polymorphisms while others carry few or none [9,11]. Within genomes of individual species, inversions may be preferentially found on particular chromosome arms. These observations may explain the differences in rates of chromosome reshuffling during the evolution of different lineages and among chromosome arms in the same lineage [27,28,40]. Even on a given arm, breakpoints are not distributed randomly; there are "hotspots" of chromosomal rearrangement and "reuse" of breakpoints in the same chromosomal region [26-30]. The frequently observed association of repetitive DNA with inversion breakpoints has suggested a general mechanism for the origin of inversions involving ectopic recombination between repeated sequences (segmental duplications and/or transposable elements). Accordingly, one factor contributing to the nonrandom distribution of inversions might simply be their nonrandom generation, subject to the presence and position of repetitive DNA on a given chromosome and in a given lineage. Another possibility, not necessarily mutually exclusive, is that only inversions with breaks at certain sites are retained in populations [9]. Coluzzi *et al. *[11] have emphasized the latter hypothesis to explain the pattern observed in *An. gambiae*: "This nonrandom pattern of inversion distribution strongly suggests that these rearrangements are the product of selection." (p. 1415).

The data gathered in this study from 82 *An. gambiae *RCIs may shed light on this question, as the nonrandom pattern of RCI distribution across the genome parallels that of fully established inversions. The question is, do the RCIs circulating in populations represent the products of selection, while others not observed (presumably including those on chromosomes 3 and X) were lost, or does this sample of RCIs reflect their nonrandom generation on 2R? We assume that immediately after their origin, most inversions are lost due to drift regardless of breakpoint location, and the probability of detection of these nascent inversions during our sampling efforts is negligible. Those that escape immediate loss are present in the population in low copy number (as RCIs), and are in a transitional ("establishment") phase which can end in maintenance or loss due to selection and/or drift [9]. Under the assumption that the 17 RCIs that were sampled repeatedly across years (in some cases nearly a decade, *e.g. *2R-17) and/or across relatively large geographic areas (*e.g. *2R-3, -5, -12, -41, -64) are identical by descent, it is likely that they are old enough to have become more common had they been the targets of strong positive selection, given at least 12 generations per year, relatively short flight range, and large effective population sizes for this mosquito [41]. That their frequencies did not apparently increase in time or space suggests that they are unlikely targets of strong selection. Other observations are consistent with this view. Whereas the number of common inversions observed in different ecological or geographic parts of the species range may differ significantly, such is not the case for RCIs. The RCI data suggest that the probability of origin and early establishment of inversions is the same across the range of *An. gambiae *(and likely, its sibling species *An. arabiensis*). The difference in outcomes between the geographic distribution of RCIs and common inversions probably reflects the role of selection in the maintenance of common inversion polymorphisms in heterogeneous environments, a later stage that RCIs may (or may not) reach.

Can the nonrandom forces responsible for RCI origin be attributed to unequal density of repetitive DNA across chromosome arms? If so, we would predict a higher density of repetitive DNA on chromosome 2R. Prior to the availability of the complete genome sequence of *An. gambiae*, the distribution of four transposable elements (two non-LTR retrotransposons, a DNA transposon and a MITE) were studied by in situ hybridization to the polytene chromosomes of the PEST strain [42]. Elements were found to be concentrated in centromeric heterochromatin and centromere-proximal euchromatin, and underrepresented at the distal ends of chromosome arms. There was a greater than expected coincidence of hybridization sites between element types. However, the observed number of sites on 2R for all four elements did not exceed that expected under a uniform distribution. Complete genome sequence of the PEST strain later revealed that overall transposon densities (~40 types were studied) indeed differed by arm [43], but did so in a pattern opposite to that observed for RCIs. Specifically, transposon density was highest on the – (59 per Mb), lower on 3L, 3R and 2L (48, 47, and 46 per Mb, respectively) and lowest on 2R (37 per Mb). Accordingly, the distribution of RCIs in natural populations is not obviously related to the distribution of transposons in the *An. gambiae *reference genome, reminiscent of the situation in some *Drosophila *lineages [27]. However, caution should be applied in interpreting these results, as it is not clear that the distribution of transposons in the PEST strain is a good reflection of their distribution in the natural populations within which the RCIs arose, especially given that PEST is chromosomally standard (uninverted) with respect to all common inversions. Segmental duplications, a second type of substrate for ectopic recombination, were detected in the reference genome, and although they seem to be less frequent than they are in mammalian genomes [43], they may comprise as much as 11% of the *An. gambiae *genome sequence [44]. Unfortunately, the spatial distribution of this class of repeated sequence has yet to be analyzed in *An. gambiae*. As a result, the relationship between RCI distribution and either transposon or segmental duplication density remains uncertain. Despite these uncertainties, the absence of a correlation between transposon distribution and breakpoint distribution in the *An. gambiae *reference genome hints that consideration should be given to an alternative model for inversion generation, one that invokes recurrent staggered chromosomal breaks in structurally unstable genomic regions as the primary cause [30]. If this mechanism predominates in *Anopheles *as it seems to in *Drosophila*, it suggests that regions of chromosome 2R are especially prone to breakages.

## Conclusion

Are RCIs evolutionarily significant? What seems clear is that common polymorphic inversions are often subject to strong positive or balancing selection. It has been argued that *An. gambiae *owes its ecological flexibility – its ability to exploit environmental heterogeneities – to its common inversion polymorphisms [11]. Here we have suggested that RCIs are not likely targets of strong positive (or balancing) selection in present-day populations of *An. gambiae*. Under unperturbed ecological conditions, they may persist for many generations as selectively neutral or nearly neutral chromosomal mutations. There is precedence for RCIs and their persistence at low frequencies in populations of *Drosophila pseudoobscura *[45]. These authors concluded that the persistence of RCIs represents a mechanism by which *D. pseudoobscura *populations store genetic variability, likening it to a sponge storing water, as suggested by Chetverikov [46]. Similarly in *An. gambiae*, RCIs represent a significant and previously unrecognized source of standing and possibly cryptic genetic variation that could produce a beneficial phenotype in response to environmental perturbation or change. Their presence at low but detectable levels across the range of *An. gambiae *testifies to the appreciable ongoing rate of chromosomal mutation and the considerable evolutionary potential hidden within *An. gambiae *populations.

## Authors' contributions

MP participated in conceiving and designing the study, chromosomal processing and analysis, statistical analysis, and drafting the manuscript. BC participated in designing the study, field collections, chromosomal processing and analysis, statistical analysis, and helped to draft the manuscript. FS participated conceiving and designing the study, field collections, and helped to draft the manuscript. MADD participated in chromosomal processing and analysis. MC participated in conceiving the study, field collections, chromosomal processing and analysis. ADT participated in designing the study and helped to draft the manuscript. CC participated in conceiving and designing the study, statistical analysis and drafting the manuscript. NJB participated in conceiving, designing and coordinating the study, statistical analyses and drafting the manuscript. VP participated in conceiving and designing the study, chromosomal processing and analysis, statistical analysis, and drafting the manuscript.

All authors read and approved the final manuscript.

## Supplementary Material

Additional file 1 **Table S1**. Distribution of rare chromosomal inversions (RCIs) in population samples of *Anopheles gambiae *sensu stricto from 1975 to 2006. Click here for file

Additional file 2 **Figure S1**. Cytogenetic map of the *Anopheles gambiae *sensu stricto ovarian polytene complement indicating numbered divisions, lettered subdivisions, and ten infradivisions per subdivision (vertical lines). Click here for file

Additional file 3 **Table S2**. Cytogenetic positions of rare chromosomal inversions (RCIs) observed in this study, and the carrier's karyotype(s) (with respect to common inversions). Click here for file
