# Supplementary material for: Chromosomal plasticity and evolutionary potential in the malaria vector Anopheles gambiae sensu stricto: insights from three decades of rare paracentric inversions
Source: BMC Evol Biol. 2008 Nov 10;8:309. doi: 10.1186/1471-2148-8-309 (PMC2654565; doi:10.1186/1471-2148-8-309)
Supplement: Additional file 1 — Table S1. Distribution of rare chromosomal inversions (RCIs) in population samples of Anopheles gambiae sensu stricto from 1975 to 2006. [file 1471-2148-8-309-S1.rtf]

Table S1. Distribution of rare chromosomal inversions (RCIs) in population samples of Anopheles gambiae sensu stricto from 1975 to 2006.

Country/Site	
Longitude	
Latitude	
Year	Sample
Size	
Observed RCIs (absolute frequency)	
The Gambia	
   Mandina Ba	16° 44' W	13° 32' N	1980	1028*	2R-9(1); 2R-10(1); 2R-11(1); 2R-12(1); 3R-2(1)	
	“	“	1981	67*	2R-18(1); 2L-4(1)	
   Jibora Kuta	16° 42' W	13° 24° N	1979	133	2L-4(1)	
   Brefet	16° 23' W	13° 14' N	1979	145	2R-9(1); 2R-10(1); 3R-1(1)	
   Sare Samba Sowe	16° 3' W	13° 37' N	2006	162	2R-67(1)	
   Ker Madi	15° 48' W	13° 38' N	1981	232	2R-10(1)	
   Sankuli Kunda	14° 46' W	13° 31' N	1979	48	2R-3(1)	
   Touba Tafsir	14° 12' W	13° 22' N	2005	24	2R-53(1)	
   Limbambulu Yamadou	14° 10' W	13° 38' N	2006	168	2R-39(2)	
   Maraban Panda	na	na	1976	na	2R-8(1)	
   na	na	na	na	na	2L-5(1)	
Guinea Bissau	
   Biquedje	15° 35' W	11° 52' N	1992	19	2R-41(1)	
   Bissau	15° 35' W	11° 52' N	1981	30	2R-16(1)	
   Beli	14° 6' W	12° 7' N	1992	46	2R-35(1)	
   Gabu	14° 6' W	12° 7' N	1982	39	2R-12(1**); 2R-20(1**)	
   Lugadjole	14° 6' W	12° 7' N	1982	50	2R-12(2)	
   Pataqui	14° 6' W	12° 7' N	1992	8	2R-39(1)	
Senegal	
   Wassadou	14° 10' W	12° 45' N	2006	18	2R-61(1)	
   Hamdalaye Pont	13° 40' W	13° 46' N	2006	43	2R-39(1*); 2L-9(1*); 2L-10(1)	
   Jingoreh Maffy	13° 40' W	13° 46' N	2006	23	2R-39(1)	
   Silling	12° 16' W	12° 32' N	2006	33	2R-39(1)	
   Samecouta	12° 11' W	12° 33' N	2006	78	2R-39(8)	
Guinea Conakry	
   Labé	12° 35' W	11° 16' N	1989	3	2R-35(1)	
   Sombili	12° 35' W	11° 16' N	1989	43	2R-35(8)	
	“	“	1990	43	2R-35(5)	
   Timbi Madina	12° 32' W	11° 12' N	1990	14	2R-35(2)	
Mali	
   Tinko	10° 45' W	13° 30' N	1984	58	2R-26(1)	
   Founia-Tintiba	10° 30' W	13° 20' N	1984	84	2R-25(1)	
   Senala	8° 16' W	12° 25' N	1982	104	2R-19(1)	
   Farada	8° 12' W	12° 26' N	1982	58	2R-21(1)	
   Kambila	8° 6' W	12° 48' N	1989	20	2R-19(1)	
   Banambani	8° 2' W	12° 48' N	1985	619	2R-26(1); 2R-27(1); 2R-31(1)	
	“	“	1989	54	2R-26(1)	
	“	“	1995	na	2R-46(1); 2R-47(1); 2R-12(1)	
	“	“	2000	na	2R-51(1)	
   Moribabougou	7° 52' W	12° 41' N	1994	334	2R-43(1); 2R-66(1)	
	“	“	1996	na	2L-7(1)	
   Ouakoro	6° 41' W	12° 36' N	1981	8	2R-15(1)	
   Banankoroni	6° 27' W	13° 18' N	1981	64	2R-13(1)	
   Douna	5° 40' W	10° 55' N	1983	131	2R-23(1)	
   Sevaré	4° 6' W	14° 31' N	1981	7	2R-14(1)	
Burkina Faso	
   Bama	4° 25' W	11° 22' N	1984	213	3R-3(1)	
   Vallée du Kou	4° 25' W	11° 22' N	1981	624	2R-17(1)	
	“	“	1985	470	2R-28(1); 2R-64(1); 2L-6(1)	
   Dioulassoba	4° 18' W	11° 11' N	1986	26	2R-62(1)	
   Soumousso	4° 3' W	11° 1' N	1981	57	2R-62(1)	
   Tanghin	2° 32' W	12° 22' N	1984	36	2R-17(1)	
   Pabre	1° 34' W	12° 29' N	1984	56	2R-24(2)	
   Ouagadougou	1° 32' W	12° 22' N	1989	na	2R-37(1)	
   Nioko	1° 23' W	11° 28° N	1989	133	2R-36(1)	
   Noungou	1° 3' W	12° 44' N	1990	78	2R-17(1)	
   na	na	na	1989	na	2R-63(1)	
Ghana	
   Navrongo	1° 5' W	10° 53' N	1992	180	2R-38(1)	
Togo	
   Kantindi	0° 17' E	10° 54' N	1987	136	2R-19(1)	
Benin	
   Serou	1° 40' E	9° 42' N	1986	89	2R-64(1)	
   Kpassa	2° 40' E	9° 17' N	1986	50	2R-5(2)	
   Tounga	3° 23' E	11° 51' N	1986	301	2R-30(1)	
Nigeria	
   Itagbe	3° 42' E	8° 21' N	1977	na	2L-3(1)	
   Sapele	5° 41' E	5° 54' N	1978	na	2R-7(1)	
	“	“	na	na	2R-1(1)	
   Old Turunku	7° 42' E	10° 49' N	1977	na	2L-2(1)	
   Turunku	7° 42' E	10° 49' N	na	na	2R-5(1)	
   Keffi area	7° 52' E	8° 51' N	1977	na	2R-6(1)	
   Matsena	10° 3' E	13° 8' N	1975	na	2R-4(1); 2L-1(1)	
   na	na	na	na	na	2R-2(1); 2R-3(1)	
Sao Tome	
   Riboque	6° 36' E	0° 12' N	1997	14	2R-48(1)	
Cameroon	
   Dombé	9° 56' E	2° 57' N	2005	2	2R-58(1)	
   Nlozok	10° 30' E	2° 51' N	2005	30	2R-59(1)	
   Ebebda	11° 16' E	4° 21' N	2000	20	2R-49(1)	
   Nkolbisson	11° 27' E	3° 52' N	1992	64	2R-40(1)	
   Bankim	11° 29' E	6° 5' N	2001	24	2R-49(1)	
   Abang	11° 31' E	3° 24' N	2005	16	2R-58(1)	
   Obala	11° 31' E	3° 52' N	2000	14	2R-50(1)	
   Odza	11° 31' E	3° 48' N	2005	25	2R-58(1)	
   Yaoundé	11° 31' E	3° 52' N	1987	11	2R-32(1)	
   Tibati	12° 36' E	6° 29' N	2001	15	2R-42(4)	
	“	“	2002-3	217	2R-49(4); 2R-52(4)	
	“	“	2005	87	2R-58(1)	
   Malarba Beka/Febadi	13° 4' E	6° 36' N	2005	9	2R-58(1); 3L-1(1)	
   Lainde Mbana	13° 30' E	9° 7' N	2005	14	2R-55(1)	
   Ndjola Kaesiki	13° 30' E	9° 5' N	2005	16	3R-4(1)	
   Sanguere Ngal	13° 30' E	9° 15' N	2005	12	2R-54(1)	
   Djamboutou	13° 31' E	8° 35' N	2005	13	2L-8(1)	
   Mayo Djarani	13° 33' E	7° 18' N	2005	12	2R-57(1); 2R-65(1)	
   Djet	13° 35' E	7° 49' N	2005	16	2R-60(1)	
   Mayo sala	13° 39' E	8° 30' N	2005	11	2R-58(1)	
   Sakdje	13° 39' E	8° 16' N	2005	8	2R-41(1)	
   Bouk	13° 48' E	8° 21' N	2005	11	2R-56(1)	
   Rompo	13° 57' E	9° 46' N	2005	4	2R-49(1)	
Central African Rep.	
   Bangouma	21° 38' E	5° 38' N	1993-4	105	2R-42(15)	
Uganda	
   Bumaga	30° 12' E	0° 52' N	1994	na	2R-45(1)	
   Sempaya	30° 12' E	0° 52' N	1994	na	2R-44(1)	
Kenya	
   Kisian	34° 45' E	0° 6' S	1987	22	2R-33(1)	
   Kisumu	34° 45' E	0° 6' S	na	na	2R-34(1)	
Mozambique	
   Fudimane	na	na	1983	na	2R-22(1)	
RCIs in bold are found in multiple years and/or multiple sites.
na = not available
* minimum sample size
** two RCIs in one specimen
