# Supplementary material for: Chromosomal plasticity and evolutionary potential in the malaria vector Anopheles gambiae sensu stricto: insights from three decades of rare paracentric inversions
Source: BMC Evol Biol. 2008 Nov 10;8:309. doi: 10.1186/1471-2148-8-309 (PMC2654565; doi:10.1186/1471-2148-8-309)
Supplement: Additional file 3 — Table S2. Cytogenetic positions of rare chromosomal inversions (RCIs) observed in this study, and the carrier's karyotype(s) (with respect to common inversions). [file 1471-2148-8-309-S3.rtf]

Table S2. Cytogenetic positions of rare chromosomal inversions (RCIs) observed in this study, and the carrier's karyotype(s) (with respect to common inversions).
RCI code	telomeric breakpoint	centromeric breakpoint	% genome	Karyotypea	
2R-1	13A9	14E9	4.6	na	
2R-2	13D5	14C7	2.1	na	
2R-3	13E9	14E6-15A1	2.5	na	
				-2---/2	
2R-4	14E9	17B6	6.0	na	
2R-5	9C2	10A0	0.6	-----/2	
				-222-/2	
2R-6	8C0	9A3	2.0	na	
2R-7	12C7-12C9	13A9	1.9	-----/-	
2R-8	13D5	14D7	2.1	-2---/2	
2R-9	13A0	15E0	7.2	-----/-	
				na	
2R-10	16A4	17B9	3.1	-1-1-/1	
				---1-/-	
				---2-/2	
				1--2-/1	
2R-11	11B6	15E1	11.1	---1-/-	
2R-12	16A8	17B7	2.8	---2-/2	
				12-2-/-	
				-1-2-/1	
				---2-/2	
				-1-1-/1	
2R-13	16D0	18C0	3.7	-11-1/2	
2R-14	17A0	18B0	2.3	-11-1/2	
2R-15	18A3	18D9	2.3	----2/2	
2R-16	9A7-9B4	13E4	9.9	---2-/2	
2R-17	8D3	10A6	3.1	-22--/2	
				-----/2	
				-11--/2	
2R-18	11B5-12E8	14C8-14D2	6.2	-2---/2	
2R-19	13D9	17C2	9.4	-11-1/1	
				-2---/2	
				-2---/2	
2R-20	7A7-7B2	12D5-12D7	11.6	12-2-/-	
2R-21	14D0	16A3-16A6	4.0	-2---/2	
2R-22	11B6	16A5-16B2	12.2	-----/1	
2R-23	8C9	9B5	2.2	-22--/2	
2R-24	7A5	7C5	1.1	-22--/2	
2R-25	15E9	16D5	2.0	-1111/2	
2R-26	10C8	12B9	3.2	12-1-/2	
				122-2/2	
				121-1/2	
2R-27	16B3	17C6	3.1	12---/na	
2R-28	9B8	15E2	14.5	-11-1/na	
2R-29	13C8	14A8	1.4	-2---/2	
2R-30	17A0	18B5	2.6	-22--/2	
2R-31	11B6	12A0	0.7	121-1/2	
2R-32	14D0-14D7	15D7-15E5	2.9	-----/1	
2R-33	14B8	17B4	7.6	-----/1	
2R-34	15E5	16D5	2.3	-1---/2	
2R-35	16A7	17A2	2.0	12-1-/1	
				1--2-/2	
				12-2-/2	
				-1-1-/2	
				1-111/1	
				-1111/2	
				-1111/1	
				1-111/-	
				-1-1-/1	
				11-1-/1	
				1--2-/2	
2R-36	9C4	11C6	3.4	-22--/2	
2R-37	8D8	9C0	2.5	----1/2	
2R-38	8C5-8C9	9C4	2.9	-----/1	
2R-39	16B0	17B7	2.7	-1-2-/-	
				12-2-/2	
				---2-/2	
				12-1-/2	
				11-1-/2	
				-1111/-	
				1111-/-	
				-1111/2	
				1111-/2	
				12-1-/2	
				21-1-/2	
				12111/1	
				12-2-/1	
				22-1-/2	
2R-40	13A0	13C8	1.5	-----/-	
2R-41	14A0	15A2	2.6	---2-/-	
				-21--/1	
2R-42	14E7	17B9	6.2	-----/-	
				-----/1	
				-----/2	
				-----/2	
				-11--/-	
				-11--/1	
				-11--/2	
2R-43	15C8	16B0	2.1	212-2/2	
2R-44	14E7	16C5	4.3	-----/-	
2R-45	15D3	17C9	5.2	-----/-	
2R-46	9C6	11B1	2.6	-11-1/-	
2R-47	8C9	10C3	4.1	-2---/2	
2R-48	13D8	14D9	2.7	-----/-	
2R-49	15A0	16B3	3.5	-----/-	
				-1---/-	
				-11--/1	
				-1---/1	
2R-50	13D5	15A2	3.4	-----/-	
2R-51	12A3	14B0	6.0	-2---/2	
2R-52	15A0	16D0	4.5	-----/-	
				-11--/1	
				-11-1/1	
				-22--/2	
2R-53	7A2	7C5	1.3	-1-1-/2	
2R-54	13A9	15C2	5.6	1222-/1	
2R-55	9C4	10D8	2.1	-211-/2	
2R-56	7A6	8C3	2.6	-2---/2	
2R-57	13C8	15D2	2.8	-2---/2	
2R-58	13D5	15D2	2.5	-1---/-	
				-1---/-	
				-2---/-	
				-2---/2	
				-1---/-	
				-1---/1	
2R-59	14E6	16A0	3.0	-----/-	
2R-60	14D0	15E7	3.5	-1---/2	
2R-61	10D6	12D3	3.8	22-2-/1	
2R-62	13E9	17B0	8.1	-2---/2	
				-1---/2	
2R-63	8D5	10B5	3.3	-22--/2	
2R-64	13D9	17B7	9.1	-2---/2	
				-2---/2	
2R-65	14E6	17B6	6.1	-1---/-	
2R-66	7B8	9A3	3.7	2-2-2/2	
2R-67	12C7	14C0	5.0	---2-/1	
2L-1	22C6	21E7	2.6	-211-/2	
2L-2	22E5	22A7	2.5	-211-/2	
2L-3	24C2-24B4	23B5-23B1	2.8	-2---/-	
2L-4	26D3-26C8	25B8	2.7	---2-/2	
				-2-1-/2	
2L-5	28B9-28B4	27D2-27C5	1.5	na	
2L-6	24C7-24D1	22A6-21F1	8.0	-22--/2	
2L-7	22C0	21D1	3.0	-11-1/2	
2L-8	26C6	25B9	2.5	-2---/2	
2L-9	22F1	22D1	1.2	---2-/2	
2L-10	22E9-22E5	22C4-22B9	1.6	---2-/2	
3R-1	34C0-34C6	37A5	5.2	-----/-	
3R-2	35B2	36C0	2.4	---2-/2	
3R-3	33D9	35B3	2.8	-11-1/2	
3R-4	32A4	33B0	2.4	-221-/2	
3L-1	46A8	44B6	3.5	-2---/2	
Common inversions	
2Rj	7C5	10C7	0.3	
2Rb	11C0	13A0	0.1	
2Rc	13A1	14A0	0.1	
2Rd	14A0	16B0	0.2	
2Ru	14A0	14E6	0.1	
2Rbk	12D4	15C0	8.5	
2La	27A0	23A0	0.3	
aPolytene chromosome configuration, after Touré et al. 1998.  The '-', '1' and '2' indicate standard homozygote, heterozygote and inverted homozygote with respect to inversions 2R j, b, c, d, u and (followed by '/') 2La.
Bold denotes RCIs detected in multiple specimens.
na = not available
